# Pattern and determinants of COVID-19 infection and mortality across countries: An ecological study

**DOI:** 10.1016/j.heliyon.2021.e07504

**Published:** 2021-07-08

**Authors:** Noha Asem, Ahmed Ramadan, Mohamed Hassany, Ramy Mohamed Ghazy, Mohamed Abdallah, Mohamed Ibrahim, Eman M. Gamal, Shaimaa Hassan, Nehal Kamal, Hala Zaid

**Affiliations:** a Department of Public Health, Faculty of Medicine, Cairo University, Egypt; b Ministry of Health and Population, Egypt; c Department of Data Science and Medical Information, DataClin CRO, Egypt; d Department of Applied Statistics, Faculty of Postgraduate Studies for Statistical Research, Cairo University, Egypt; e Tropical Health Department, High Institute of Public Health, Alexandria University, Egypt; f Medical Research Division, National Research Center, Giza, Egypt; g Department of Research, Children's Cancer Hospital (CCHE) 57357, Egypt; h Department of Medical Biochemistry and Molecular Biology, Faculty of Medicine, Cairo University, Egypt

**Keywords:** Social mobility, Ecological factors, COVID-19 mortality, COVID-19 transmissibility, Exponential model, Linear model, COVID-19 incidence, COVID-19

## Abstract

**Background:**

This work aimed to identify the mathematical model and ecological determinants of COVID-19 infection and mortality across different countries during the first six months of the pandemic.

**Methodology:**

In this study, authors used the online available data sources of randomly selected 18 countries to figure out potential determinants of COVID-19 transmissibility and mortality. The studied variables were environmental factors (daily average temperature, daily humidity), socioeconomic attributes (population age structure, count and density, human development index, per capita income (PCI), gross domestic product, internet coverage) mobility trends and chronic diseases. Researchers used the linear and exponential time series analysis, and further utilized multivariate techniques to explain the variance in the monthly increase in cases and deaths.

**Results:**

In the first two months, the R^2^ of linear models for the cases and deaths were higher than that of the corresponding R^2^ of the exponential model. Later one, R^2^ of the exponential model was occasionally relatively higher than that of the linear model. The exponential growth rate of new cases was significantly associated with mobility trends (β = 0.00398, *P* = 0.002), temperature (β = 0.000679, *P* = 0.011), humidity (β = 0.000249, *P* < 0.001), and the proportion of population aged ≥65 years (β = -0.000959, *P* = 0.012). Similarly, the exponential growth rate of deaths was significantly associated with mobility trends (β = 0.0027, *P* = 0.049), temperature (β = 0.0014, P < 0.001), humidity (β = -0.0026, P < 0.001), and PCI of countries. During this period, COVID-19 transmissibility was evident to be controlled as soon as social mobility is decreased by about 40% of the baseline over 3 months controlling for the other predictors.

**Conclusion:**

Controlling of COVID-19 pandemic is based mainly on controlling social mobility. Role of environmental determinants like temperature and humidity was well noticed on disease fatality and transmissibility. Socio-demographic determinants of COVID-19 spread and fatality included modifiable risk factors like PCI and non-modifiable risk factors like ageing.

## Teaser

Covid-19; Incidence rate; Mortality rate; Social mobility; Ecological factors; Contact frequency.

## 1 Introduction

The World Health Organization (WHO) estimated that approximately one-third (e.g., 20 million) of the annual deaths worldwide were attributed to infectious diseases. Furthermore, three of the 10-topped causes of deaths are lower respiratory tract infection, tuberculosis, and diarrheal disease, and many of these diseases can be prevented or treated for as little as one dollar for a head (Word Health Organization, 2020). The morbidity from infectious disease has increased during the past few decades and represents at least 70% of emerging infectious diseases (EID), which are a significant burden on global economic and public health (Dye, 2014; Morens et al., 2004). Emerging infectious diseases (EIDs) cause a substantial economic and public health burden in the world (Binder et al., 1999; Halliday et al., 2017). The most likely causes of the emergence of EIDs are socioeconomic, environmental, and ecological factors (Binder et al., 1999; Dye, 2014; Halliday et al., 2017; Woolhouse and Gowtage-Sequeria, 2005). It was postulated that these factors provide a clue for identifying regions where new EIDs are most likely to originate (Woolhouse and Gowtage-Sequeria, 2005). Socioeconomic, environmental, and ecological factors present a basis of risk for wildlife zoonotic and vector-borne EIDs originating at lower latitudes, where reporting effort is low (Jones et al., 2008; Woolhouse and Gowtage-Sequeria, 2005).

One of these EIDs is the severe acute respiratory syndrome coronavirus 2 (SARS-CoV-2) that causes corona virus disease 2019 (COVID-19). SARS-CoV-2 was firstly identified in December 2019 in Wuhan city, in China. It resulted in unusual pneumonia after visiting an animal market that sells poultry, fish, and other animals to the public (Xu et al., 2020). This outbreak was soon reported to the WHO. Due to this pandemic, the entire life has been changed. Millions of people have been infected (186,357,958), and hundreds of thousands have been deceased (4,026,907) by the novel coronavirus (Worldmeter, 2021). Countries adopted different strategies to combat the spread of this pandemic. Many countries implemented the national lockdown policy that extended for different duration during the daytime. Another containment strategy was social distancing and panning going out of homes without wearing facemasks. International travel bans from and to infected countries was another effective strategy used to face the spread of this pandemic (Cheng et al., 2020; Movsisyan et al., 2021; World Health Organization, 2020).

It is noteworthy that SARS-CoV-2 does not affect everyone in the same way, furthermore, it is not easy to understand the consequences or to predict how this pandemic affects differently various countries. There are several reasons that could explain why different population are affected by this pandemic in different ways. These conditions can include socioeconomic factors (income, population density, distribution of human population, urban and rural settings, education level, and lifestyle, the size of household, and homeowners & tenants), behavior factors (direct contact with domestic and wild animals, migration, social interactions), and environment factors (humidity, temperature, wind spread, climate change, deforestation, agricultural growth) (Barratt et al., 2019; Dehghani and Kassiri, 2020).

There are many published research that studied several epidemiological factors associated with SARS-CoV-2 transmission and test positivity. These studies developed statistical regression models using the online available data to predict the country-level conditions related to the COVID-19 pandemic (Karmakar et al., 2021; Kathe and Wani, 2020). Analyzing data retrieved from John Hopkin's Hospital Database,  Islam et al. (2020) found that high temperature, wind speed, and humidity were not favorable environment conditions for SARS-CoV-2 transmission. A recently published article studied the metrological factors associated with SARS-CoV-2 contagiousness and fatality across five large cities (Karachi, Lahore, Islamabad, Peshawar, and Gilgit-Baltistan) in Pakistan. Authors concluded that there was a significant positive correlation between SARS-CoV-2 transmission and all the temperature ranges (maximum, minimum and average). They also observed a negative correlation between SARS-CoV-2 transmission and humidity, diurnal temperature range and rainfall. Furthermore, they found that SARS-CoV-2 related deaths were positively associated with temperature and were negatively associated with humidity (Basray et al., 2021). Hence, this study aimed to address the simultaneous and interacting impact of sociodemographic attributes social mobility, and ecological determinants on the COVID-19 transmissibility and mortality across different countries.

## 2 Methods

### 2.1 Sample size and study setting

Considering that a total of 193 countries are members of the United Nation and 15 countries reported 0 incidence of COVID-19 by the end of June 2019. A pilot sample of 18 countries (10%) was randomly selected using a simple random sampling technique. We applied the random generation function of Microsoft Excel to select the 18 studied countries. The selected countries were Australia, Brazil, China, Canada, Egypt, France, Germany, Indonesia, Iran, Italy, Japan, Lebanon, Norway, Oman, Spain, Sweden, United Kingdom, and United States of America (USA). Of note, the selected countries represented the 6 major continents, had different climates, socioeconomic status, population densities, and numbers of confirmed COVID-19 cases and deaths.

### 2.2 Data collection

Through the period from 1^st^ of January to 30^th^ of June 2020, the daily counts of COVID-19 confirmed cases and deaths, and socioeconomic attributes for the 18 selected countries were collected from Our Word in Data website (Our World in Data, 2021). Sociodemographic attributes included population density per Km^2^, human development index (HDI), per capita income (PCI), gross domestic product (GDP) in (USD), internet coverage, population count, proportion of the population aged 65 years or above, and proportion of the population suffering from chronic diseases i.e diabetes mellitus (DM). The daily average temperature and relative humidity of the capital cities of the selected countries were collected from the Weather Underground website (Wheather Underground, 2020). The recorded policy responses of the selected countries to COVID-19 were retrieved from the International Monetary Fund (International Monetary Fund, 2021). For each of the selected countries (except for China and Iran), google daily documentation on mobility trends across different categories of places such as retail and recreation, groceries and pharmacies, parks, transit stations, workplaces, and residential was retrieved. Google documentation was presented by percent change in mobility across the mentioned places compared to the baseline, which is the median value of mobility trends for the corresponding day of the week, during 5 weeks from Jan 3 – Feb 6, 2020 (Community Mobility Reports, 2021).

### 2.3 Statistical analysis

Data analysis was conducted using Statistical Package for Social Science (SPSS) software (version 25, Chicago, USA). The Daily counts of confirmed cases and deaths for each country were split by month and were further linearly and exponentially modeled with time. The linear equation was based on the equation (y_1_ = a +βx_1_), where y is the confirmed cases or deaths for a given day of this month, the constant (a) is the number of cases at day 0 (the count before the start of that month), β is the linear slope coefficient which is presenting the average daily increase in the count of confirmed cases or deaths within this month, and x is the day number of that month. The exponential equation was based on the equation (y1 = a e^βx1^), where e^β^ is the average daily multiplying factor for increase or in other words, the exponential growth rate in each month. The daily average temperature of the capital cities was aggregated to the monthly temperature for each country. Google documentation was handled using big data analytics with R software to aggregate the data into the monthly mean percent change from the baseline across six categories of places for each of the selected countries.

The linear and exponential slope coefficients hereinafter referred to as linear growth rate and exponential growth rate, respectively. Non-parametric correlation between variables was assessed using Spearman rho's test. The monthly exponential growth rates of confirmed cases and deaths were regressed versus the collected predictors for each country using mixed-effects model with unstructured covariance. The highly correlated variables were subjected to principal component analysis (PCA) with varimax rotation, and the extracted PCs were utilized for the regression models.

### 2.4 Ethical considerations

This study was exempt from review by the Ethics Committee of the Ministry of Health and Population, Egypt.

## 3 Results

### 3.1 The mathematical pattern of incidence and mortality at the start of the pandemic

Time series analysis was conducted for the monthly records of COVID-19 confirmed cases and deaths, both linear and exponential growth models were studied. The R^2^, constant, and β (the slope coefficient) of the linear and exponential models are presented in (Supplementary material 1). In January 2020, China was the only country that reported confirmed deaths due to SARS-CoV-2 infection. China, Australia, Canada, France, Germany, and USA have reported confirmed cases with COVID-19 that were valid for statistical modeling. During January, the R^2^ of linear models for confirmed cases by time in most of the selected countries was higher than that of the corresponding R^2^ of the exponential model for the same data, in other words, the growing data for confirmed cases were better described with linear equations.

### 3.2 Further mathematical pattern of new cases and mortality

**Figure 1:**
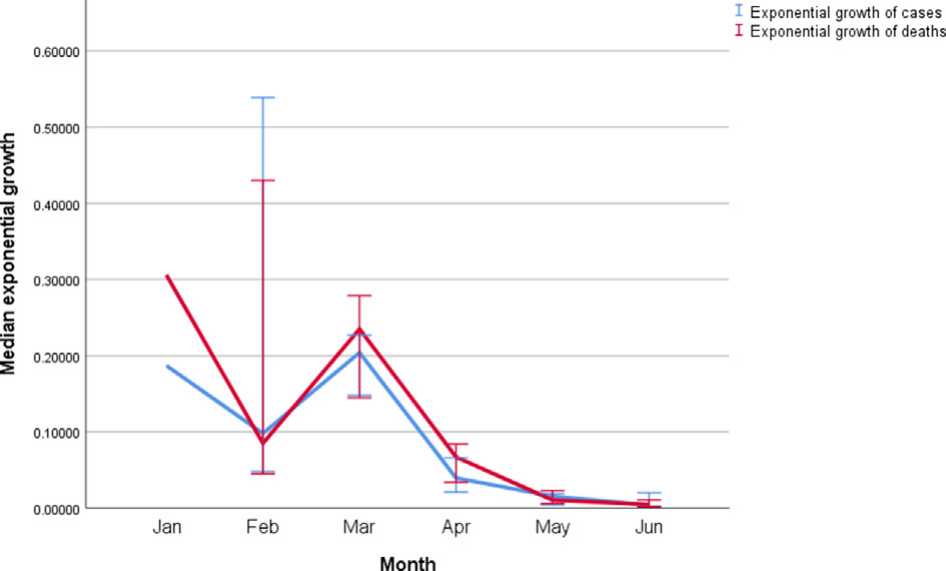
Median monthly exponential growth rate of confirmed cases and deaths.

**Figure 2:**
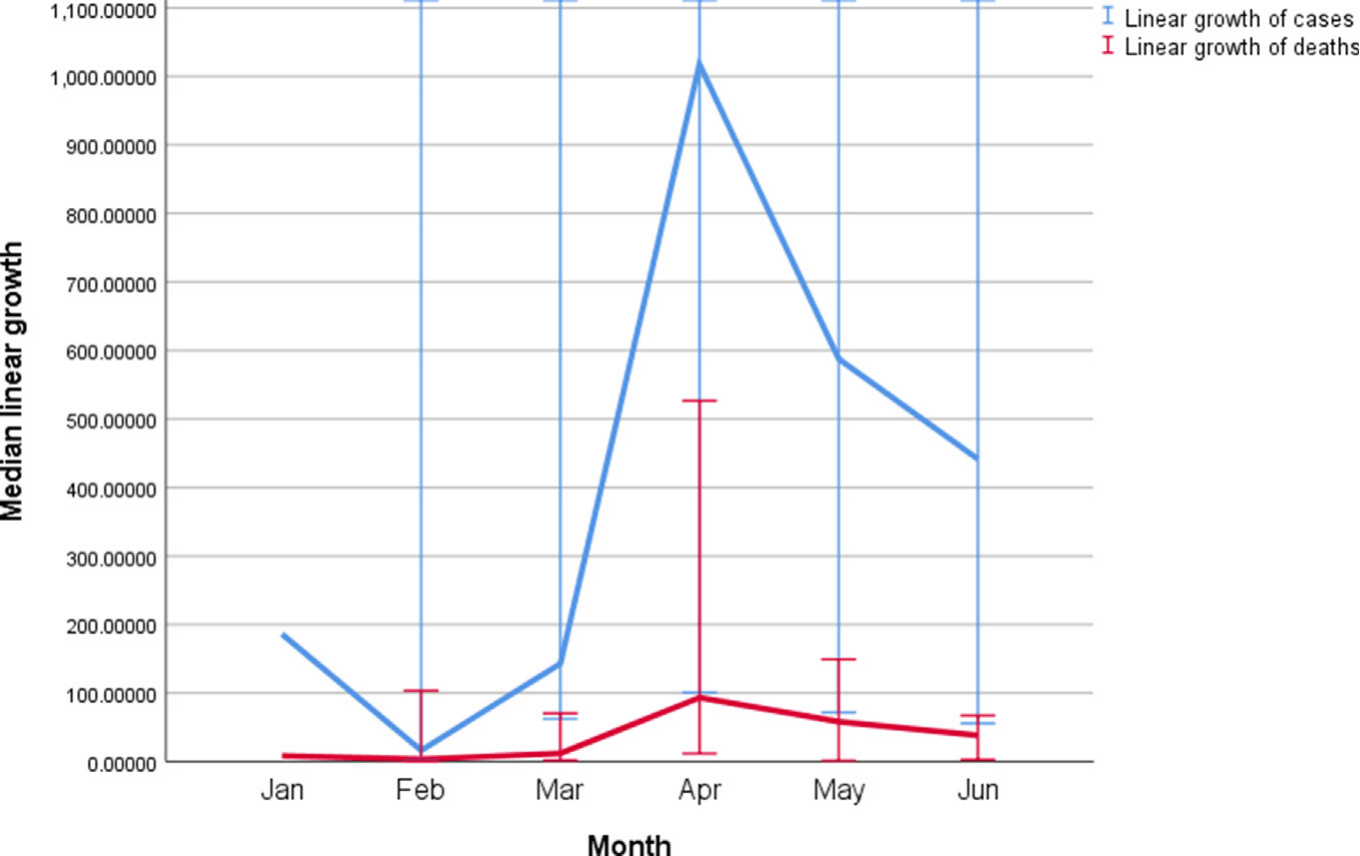
Median monthly linear growth rate for confirmed cases and deaths.

Starting from February 2020, and except for Egypt, Indonesia, and Brazil, all the selected countries started to report an increasing number of confirmed COVID-19 cases. Until June 2020, the R^2^ of the exponential models were occasionally relatively higher than that of the linear models for confirmed COVID-19 cases and deaths, while in most models they were nearly equal. The monthly exponential growth rates of confirmed cases and deaths were perfectly correlated (Spearman correlation coefficient = 0.945, *P* < 0.01). For the 18 countries, the median monthly growth rates of confirmed cases and deaths are presented in (Figures 1 and 2), respectively.

### 3.3 The pattern of mobility and its effect on COVID-19 new cases and mortality

**Figure 3:**
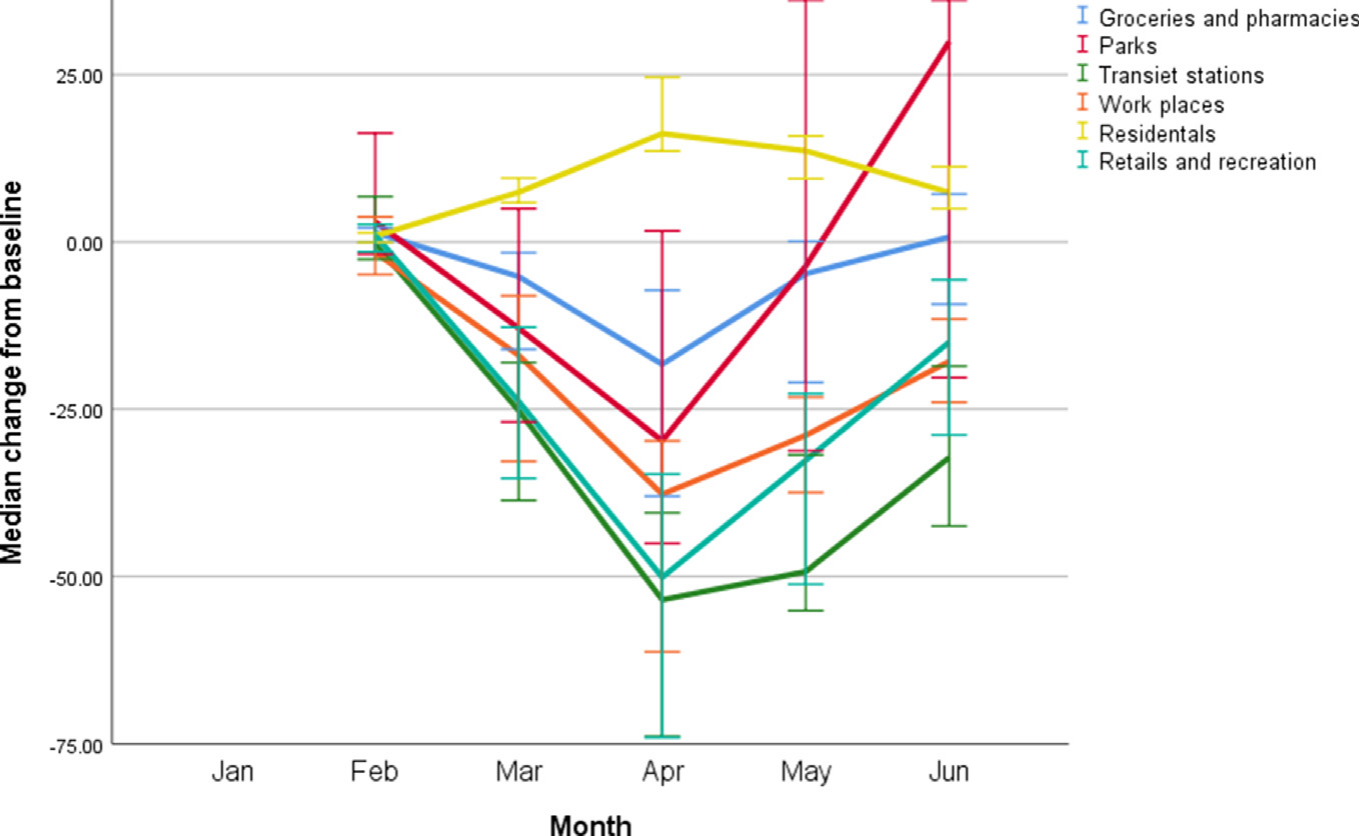
Median change in mobility trends from baseline based on Google mobility reports.

The monthly change in mobility trends from baseline across different place categories is presented in (Figure 3). In February 2020, mobility trends almost did not change from the baseline, then the median value started to decrease in March and April for all place categories except for mobility trends towards residential places. Afterward, an inflection has occurred and the median values for mobility trends increased towards all place categories until the final time point in June 2020, in which most of the median values were returned to the baseline. Interestingly, the median value of mobility trends toward parks, beaches, and public gardens has even exceeded the baseline in June 2020 (+25%).

It is worth noting that changes in mobility trends affected the exponential growth of COVID-19 new cases and deaths in the next month. So, we can notice a decrease in mobility trends from baseline in March, whose median exponential growth is the highest for cases and deaths, but we can further notice a decrease in exponential growth in the next month of April. This is because the confirmed cases usually get infected in a given month and been reported in the next month after a lag period of incubation and confirmation of infection. This remark was tested by assessing the correlation between the exponential growth rates and mobility trends in the same month. The results are presented in (Table 1), most of the correlations were week and insignificant, while when mobility trends were tested for correlation with the exponential growth rates of the next month, we got significant strong positive correlations for new cases and deaths (Table 2).

**Table 1:** Correlations between changes in mobility trends toward six categories of places with exponential growth rates of cases and deaths of the same month.

Correlations										
			Cases_Exp growth	Deaths_Exp growth	grocery and pharmacy	Parks	Transient stations	Workplaces	Residential	Retail and recreation
Spearman's rho	Cases_ Exp growth	Correlation Coefficient	1.000	.945∗∗	-0.047	-.319∗∗	.244∗	.311∗∗	-0.187	0.078
		Sig. (2-tailed)	-	<0.001	0.684	0.005	0.032	0.006	0.103	0.502
	Deaths_ Exp growth	Correlation Coefficient	.945∗∗	1.000	-0.183	-.345∗∗	0.077	0.070	0.048	-0.121
		Sig. (2-tailed)	<0.001	-	0.142	0.005	0.541	0.576	0.699	0.335

∗∗ Correlation is significant at the 0.01 level (2-tailed).

∗ Correlation is significant at the 0.05 level (2-tailed).

**Table 2:** Correlations between changes in mobility trends with exponential growth rates of cases and deaths of the next month.

Correlations						
			Cases_Linear growth	Cases_Exp growth	Deaths_Linear growth	Deaths_Exp growth
Spearman's rho	Grocery and pharmacy	Correlation Coefficient	-0.113	.452∗∗	-0.193	.495∗∗
		Sig. (2-tailed)	0.376	<0.001	0.129	<0.001
	Parks	Correlation Coefficient	-0.039	0.110	-0.057	0.173
		Sig. (2-tailed)	0.762	0.385	0.656	0.174
	Transient stations	Correlation Coefficient	-0.080	.712∗∗	-0.105	.748∗∗
		Sig. (2-tailed)	0.530	<0.001	0.411	<0.001
	Workplaces	Correlation Coefficient	-0.184	.770∗∗	-.253∗	.775∗∗
		Sig. (2-tailed)	0.147	<0.001	0.046	<0.001
	Residential	Correlation Coefficient	0.240	-.727∗∗	.284∗	-.744∗∗
		Sig. (2-tailed)	0.056	<0.001	0.024	<0.001
	Retail and recreation	Correlation Coefficient	-0.212	.688∗∗	-.282∗	.701∗∗
		Sig. (2-tailed)	0.093	<0.001	0.025	<0.001

∗∗ Correlation is significant at the 0.01 level (2-tailed).

∗ Correlation is significant at the 0.05 level (2-tailed).

### 3.4 Effect of mobility on the number of newly diagnosed COVID-19 cases

The monthly lagged exponential growth rates of new cases showed an intermediate positive correlation with mobility trends to glossaries and pharmacies (r = 0.452, P < 0.01), and a strong positive correlation with MPC in mobility trends to transit stations (r = 0.712, P < 0.001), mobility trends to workplaces (r = 0.77, P < 0.01), and mobility trends to retail and recreation (r = 0.688, P < 0.01), while it showed strong negative correlation with mobility trends to residential places (r = -0.727, P < 0.001).

### 3.5 Effect of mobility on the COVID-19 related mortality

The monthly lagged exponential growth rate of deaths showed an intermediate significant positive correlation with the change in mobility trends to groceries and pharmacies (r = 0.495, P < 0.01), while it had a strong positive correlation with the change in mobility trends to retail and recreation places (r = 0.7, P < 0.01), change in mobility trends to workplaces (r = 0.775, P < 0.01), and change in mobility trends to transit stations (r = 0.748, P < 0.01), and it showed a strong negative correlation with change in mobility trends to residential places (r = -0.744, P < 0.01).

### 3.6 Effect of temperature, humidity and sociodemographic factors on COVID-19 new cases, and deaths

The monthly average temperatures of countries had a weak significant negative correlation with exponential growth rates of deaths in the next month (r = -0.25, P = 0.038), while it did not significantly correlate with exponential growth rates of the number of new cases (-0.08, P = 0.49). Relative humidity was significantly correlated with the exponential growth rates of the number of newly confirmed cases (r = 0.28, P = .017) and deaths (r = 0.31, P = 0.008) of the next month (Table 3). On the other hand, researchers could not get any significant correlations upon testing the overall exponential growth rates for the number of newly diagnosed cases and deaths with the socioeconomic factors of the selected countries.

**Table 3:** Correlations between average temperature and humidity with exponential growth rates of new cases and deaths of the next month.

Correlations						
			Cases_Linear growth	Cases_Exp growth	Deaths_Linear growth	Deaths_Exp growth
Spearman's rho	Monthly average temp	Correlation Coefficient	-0.131	-0.083	-0.172	-.247∗
		Sig. (2-tailed)	0.271	0.490	0.152	0.038
	Monthly average humidity	Correlation Coefficient	-0.077	.280∗	-0.043	.314∗∗
		Sig. (2-tailed)	0.519	0.017	0.724	0.008

∗∗ Correlation is significant at the 0.01 level (2-tailed).

∗ Correlation is significant at the 0.05 level (2-tailed).

### 3.7 Predictors of COVID-19 transmissability and mortality

We modeled the exponential growth rates for the new cases and deaths of COVID-19 with time (months), the principal component of mobility trends of the previous month, the temperature of the previous month, the humidity of the previous month, the proportion of population aged 65 years or above, the prevalence of DM, and PCI of each country as predictors.

#### 3.7.1 Model 1: incidence rates

The exponential growth rate of newly diagnosed COVID-19 cases was significantly different across the tested four months (P < 0.001). Moreover, the variable was significantly different in all pairwise comparisons of the tested time points (P < 0.01). The intercept of the model was estimated at 0.16 (95%CI: 0.12–0.19, P < 0.01), most of the decline in the dependent variable took place in April (-0.13, 95%CI: -0.16 -0.10, P < 0.01) and the decline continued until June (-0.16, 95%CI: -0.19 -0.13, P < 0.01) matching with the monthly decline in median values presented in (Figure 1). Mobility trends were significant predictor with the highest estimate (0.00398, 95% CI: 0.00159–0.00638, P = 0.002) followed by temperature (0.000679, 95%CI: 0.000169–0.001189, P = 0.011), humidity (0.000249, 95%CI: 0.000169–0.001189, P < 0.001), and the proportion of population aged 65 years or above in each county (-0.000959, 95%CI: -0.0016- -0.000026, P = 0.012). The other determinants were statistically insignificant. The model suggests that the exponential growth rate of COVID-19 new cases would be fully controlled if mobility is decreased by about 40% of the baseline over 3 months (0.1552/0.00398) controlling for the other predictors. The model also suggests that one unit decline in mobility is equal in effect to about 6 degrees Celsius decline in temp (0.00398/0.000679) and about 16° decline in relative humidity (0.00398/0.000249) (Table 4).

**Table 4:** Estimates of fixed effects (Model 1).

Estimates of Fixed Effects (Model 1)^a^^,^^b^							
Parameter	Estimate	Std. Error	Df	t	Sig.	95% Confidence Interval	
						Lower Bound	Upper Bound
Intercept	0.155287	0.017119	26.127	9.071	<0.001	0.120106	0.190468
June	-0.163131	0.014233	16.828	-11.461	<0.001	-0.193184	-0.133078
May	-0.153123	0.014151	17.062	-10.821	<0.001	-0.182970	-0.123275
April	-0.129927	0.015765	16.189	-8.242	<0.001	-0.163315	-0.096539
March	0	0					
PC of mobility trends	0.003987	0.001174	30.060	3.397	0.002	0.001590	0.006383
Temp	0.000679	0.000248	27.274	2.733	0.011	0.000169	0.001189
Humidity	0.000249	5.921562E-05	23.021	4.200	<0.001	0.000126	0.000371
proportion of population above 65 years	-0.000959	0.000318	11.136	-3.019	0.012	-0.001657	-0.000261
Diabetes prevalence	0.000429	0.000514	14.015	0.835	0.418	-0.000673	0.001532
PCI	2.479417E-08	1.632798E-07	14.691	0.152	0.881	-3.238678E-07	3.734561E-07

a Dependent Variable: Exponential growth rate for new cases.

b Residual is weighted by Population Density (/km2).

#### 3.7.2 Model 2: COVID-19 mortality

The exponential growth rate of deaths was significantly different across different months (P < 0.01), and it was also significantly different in all pairwise comparisons of the tested time points (P < 0.01). The intercept of the model was estimated at 0.20 (CI: 0.14–0.26, P < 0.01), most of the decline in the dependent variable similarly took place in April (-0.14, 95%CI: -0.19- -0.09, P < 0.01) and the decline continued until June (-0.20, 95%CI: -0.25 -0.14, P < 0.01) matching with the monthly decline in median values presented in (Figure 1). Mobility trends had the highest estimate (0.0027, 95%CI: 0.00001–0.0054, P = 0.049) followed by temperature (0.0014, 95%CI: 0.0009–0.0019, P < 0.001), humidity (-0.0026, 95%CI: 0.0041- -0.0001, P < 0.001) and PCI of countries (-3x10^−7^, -5.72 × 10^−7^- -9.57 × 10^−8^, P = 0.009) (Table 5).

**Table 5:** Estimates of fixed effects (Model 2).

Estimates of Fixed Effects (Model 2)^a^^,^^b^							
Parameter	Estimate	Std. Error	df	t	Sig.	95% Confidence Interval	
						Lower Bound	Upper Bound
Intercept	0.199858	0.027476	18.521	7.274	<0.001	0.142249	0.257467
June	-0.195938	0.024807	15.868	-7.899	<0.001	-0.248561	-0.143315
May	-0.183183	0.024708	15.958	-7.414	<0.001	-0.235573	-0.130793
April	-0.141371	0.022041	15.503	-6.414	<0.001	-0.188218	-0.094525
March	0	0					
PC of mobility trends	0.002717	0.001309	23.276	2.076	0.049	1.127571E-05	0.005423
Temp	0.001426	0.000251	18.727	5.676	<0.001	0.000900	0.001952
Humidity	-0.000267	7.000827E-05	15.853	-3.815	0.002	-0.000416	-0.000119
proportion of population above 65 years	-8.677099E-05	0.000194	14.971	-0.448	0.660	-0.000499	0.000326
Diabetes prevalence	0.000404	0.000394	19.990	1.027	0.317	-0.000417	0.001225
PCI	-3.341040E-07	1.120112E-07	15.262	-2.983	0.009	-5.724931E-07	-9.571477E-08

a Dependent Variable: Exponential growth rate for deaths.

b Residual is weighted by Population Density (/km2).

## 4 Discussion

Unfortunately, the pandemic of COVID-19 is spreading very rapidly across the world causing large number of deaths despite the relatively low case fatality ratio. However, this is not the worst scenario, scientist warn the word from more waves of COVID-19 especially worldwide herd immunity is still away (Wise, 2020).

In this research, we tried to address the impact of different socio-demographic, mobility trend, and ecological factors that could affect the transmissibility and fatality of COVID-19. In this work researchers reported that for the first two months, the R^2^ of linear models for new cases and deaths were higher than that of the corresponding R^2^ of the exponential model. Later one, R^2^ of the exponential models were occasionally relatively higher than that of the linear models, while in most models they were nearly equal. The number of COVID-19 new cases was significantly associated with mobility trends, temperature, humidity, and the proportion of patients aged 65 years or above. Similarly, COVID-19 mortality was significantly associated with mobility trends, temperature, humidity, and PCI of countries. During this period, COVID-19 incidence was evident to be controlled as soon as social mobility is decreased by about 40% of the baseline over 3 months controlling for the other predictors.

During the first wave of the pandemic, it was so hard to predict the exact determinants of COVID-19 severity due to the large discrepancy of testing regulations and the pandemic precautionary measures between the countries. Compliance to social distancing and travel ban are important factors to be considered, which appeared in the study by Pana et al. (2021) on 38 countries which reported more than 25 COVID-19 related deaths till 8th of June 2020. They studied the association between demographic, social, environmental, and economic parameters and the mean mortality rate. The multi-variate analysis revealed that international arrivals condition was the major determinant associated with the COVID-19 death rate, while BCG vaccination,  prevalence of hypertension and testing capacity were slightly associated. The other studied parameters such as temperature, population capacity, GDP and other ones were not statistically significant (Pana et al., 2021). Similarly, Hassan et al., conducted a geospatial study to identify the ecological determinant of COVID-19 related incidence and mortality in Africa (Hassan et al., 2021). They concluded that COVID-19 incidence rate was positively associated with overcrowding, health expenditure, human immunodeficiency virus (HIV) infection and air pollution and negatively associated with BCG vaccine while, COVID-19 fatality was positively related to asthma prevalence and tobacco use.

### 4.1 Exponential versus linear growth equation

In order to identify the statistical model that clearly predict the pattern of transmissability and fatality related to COVID-19 within the earliest 6 months of the COVID-19 pandemic we randomly piloted 18 countries representing 6 continents. Until June 2020, the R^2^ of the exponential models were occasionally relatively higher than that of the linear models for confirmed COVID-19 cases and deaths, while in most models they were nearly equal. We remarked that the increase in Exp (β) of a given month implies an increase in linear (β) in the next month, so the highest calculated median Exp (β) in March implied for the highest median linear (β) in April 2020. This is because the exponential growth multiplies the counts over time, which in turn increases the absolute counts in the next months. Similarly, in a study conducted by Komarova et al. (2020), the pattern of growth was either exponential or power-law growth. They further cleared that the pattern of spread of each country depended on the time when the pandemic started; if the pandemic started early the pattern was exponential, while later pandemic following Italy was power-law pattern.

### 4.2 Social mobility trends

Many countries adopt different strategies of national lockdown despite its damaging effect on the economy and education. Mobility can be used as a proxy measure of contact frequency, so social mobility is drastically affecting the disease transmission. It is considered as one of the main control measures until the COVID-19 vaccine impact is fully evaluated. In the work presented here the exponential growth of infection was significantly correlated with the social mobility pattern of the preceded month. This is explained by the duration of the incubation period of COVID-19 and reporting was recorded in the month following. In the same line, Cartenì et al. (2020), proclaimed that both the number of tested cases, proximity to the outbreak area, and mobility of the citizen are the main predictors of COVID-19 transmission. One of the significant types of social mobility was trips; within 3 weeks trips were significantly associated with an increased COVID-19 transmission risk. Interestingly, this duration exceeded the containment duration which is 14 days. Of note, in this research, the effect of social mobility may be diluted due to the inclusion of countries with different wealthy indexes. Weill et al. (2020) reported that wealthier communities had lower social mobility than poorer communities, furthermore, the direction of mobility was more toward the least crowded areas before the pandemic than the more crowded ones and vice versa in poorer communities. In the United Kingdom, reduction of social mobility to 57.3%–65.9% of the pre-lockdown situation would decrease the reproduction rate below 1 which means pandemic control. Reports noted that social mobility contributes to 80% of disease transmissibility (Sussex, 2020). Similarly, Badr et al. (2020), reported the Pearson correlation between COVID-19 transmission and social mobility exceeded 0.7 in 25 states in USA. In this research, the effect of social mobility may be diluted due to the inclusion of countries with different wealthy indexes and studying other important environmental factors. Weill et al. (2020) reported that wealthier communities had lower social mobility than poorer communities, furthermore, the direction of mobility was more toward the least crowded areas before the pandemic than the more crowded ones and vice versa in poorer communities.

In the current study, social mobility was revealed to be the most important determinant of the incidence and virulence of COVID-19 infections. This was simultaneously controlled by the presence of the other different determinants in the same model. The mobility patterns across the studied countries were a reflection for the nationwide decisions taken for controlling the pandemic. Most of these decisions were effective by March 2020, responding to the dramatic increase in confirmed cases and deaths. Accordingly, social mobility was decreased which in turn decreased the incidence of COVID-19. However, due to several causalities, most countries decided to lessen the precautionary measures and made a gradual re-opening. Most of the re-opening decisions were effective by May and June 2020. Interestingly, we remarked an inflection in the social mobility graphs in April 2020, thus we are worried about a potential correlation with new waves of the pandemic. Interestingly, the model suggests that a 40% decrease in social mobility would control the incidence of the infections in 3 months, taking into considerations that the model was utilizing data from March to June 2020, during which the toughest decisions were taken by the governments. On the other hand, the lockdown policies don't alone prevent the transmission of the disease, but combined with other regulations like wearing face masks, using sanitizers and hand washing could be more effective in decreasing the incidence of the disease (Savaris et al., 2021).

### 4.3 Temperature and humidity

A questionable issue arose during this pandemic, whether climatic or demographic characteristics enable a more significant transmission of the virus. The Environmental temperature was thought to affect the virus survival on surfaces consequently it affects viral transmissibility. In this research, we also remarked that temperature and humidity had a significant determinant on the incidence of COVID-19, however, the effect was trivial compared to that of social mobility The result supported the first reported statistically significant relationship of negative correlation between the average environmental temperature and exponential growth rates of the newly diagnosed COVID-19 cases (Livadiotis, 2020). Similarly, Mecenas et al. (2020), reviewed the results of 517 published research articles that evaluated the effect of humidity and temperature on the viral spread. Only 17 articles fulfilled the inclusion criteria, researchers concluded that hot and wet weather significantly affect COVID-19 transmission. On the same line, Prata et al. (2020) demonstrated that there is more than 4.8% reduction in the cumulative incidence of newly diagnosed cases with each rise in temperature by 1 °C. Similarly, Basray et al. (2021), showed that for every unit increase in humidity, there was a 3.345 reduction in the daily reported cases of COVID-19, while for every unit increase in humidity, there was a noticed increase in the number of daily reported cases by 10 times. Interestingly, Anis (2020) demonstrated that the optimal temperature for virus transmission was between 13-24 °C based on analysis of data collected from March to November 2020. On the contrary, among 63 areas in China and more than a hundred locations in other seven different countries, the high temperature, humidity and ultraviolet ray exposure were not significant metrological factors associated with the COVID-19 infectivity (Pan et al., 2021).

### 4.4 Socioeconomic factors

Since the beginning of the pandemic, there was no clue on the contribution of countries’ socioeconomic attributes to the propagation of COVID-19 infection. To the best of our knowledge, no previous studies investigated these factors as determinants of transmission. In the current study, despite showing a non-significant contribution to COVID-19 contagiousness and fatality, these attributes remain of interest for future across-country studies.

## 5 Limitation and strength

To the best of our knowledge, this research is one of the first few studied that addressed the statistical pattern of the spread and mortality of COVID-19. In addition, researchers simultaneously modeled the effect of sociodemographic factors, social mobility, and ecological factors like temperature, and humidity on the number of newly diagnosed cases and deaths due to COVID-19. Studying a set of different predictors in one model makes the estimates more accurate and precise because the contribution of each predictor is controlled. An important point of limitation was that case definition of COVID-19 suspected or confirmed case may vary across countries and even may vary by time in the same country. This may affect the actual number of reported cases. The same problem can be encountered - but to a lesser extent with the number of COVID-19 deaths. These facts may affect the external validity of our models. Finally, larger studies including more countries several months after the pandemic is necessary especially after starting vaccination against SARS-CoV-2.

## 6 Conclusion

Data-driven suppositions can efficiently and proactively guide the governmental measures taken to lessen the social, health, and economic impacts of the COVID-19 pandemic. During the first six months of the pandemic, the multivariate analysis showed that the changes in mobility trends across countries dramatically affected the incidence and mortality rates across different countries, thus, it might be used as a proxy measure of contact frequency. Controlling of COVID-19 pandemic is based mainly on controlling social mobility. Role of environmental determinants like temperature and humidity was well noticed on disease fatality and transmissibility. Socio-demographic determinants of COVID-19 spread and fatality included modifiable risk factors like PCI and non-modifiable risk factors like ageing. Finally, it is necessary to conduct larger similar studies that include more countries several months after the pandemic especially after the start of vaccination era against SARS-CoV-2.

**What is already known on this subject**

Emerging literature separately investigated social mobility trends, ecological and socioeconomic factors to explain and further predict COVID-19 incidence and mortality rates across different provinces of the same country.

**What this study adds**

This study assembled social mobility trends, ecological and socioeconomic attributes in one statistical model explaining the pattern of COVID 19 realted transmissability and mortality in 18 different countries. Hence, it presents more accurate weight for each predictor, controlled for the presence of the other factors.

The model suggests that one unit decline in social mobility is equally effective in declining COVID-19's incidence to about 6 degrees Celsius decline in temperature and about 16° in relative humidity.

## Declarations

### Author contribution statement

Noha Asem: Conceived and designed the analysis; Analyzed and interpreted the data.

Ahmed Ramadan: Analyzed and interpreted the data; Wrote the paper.

Mohamed Hassany, Ramy Mohamed Ghazy, Mohamed Abdallah, Eman M. Gamal, Shaimaa Hassan, Nehal Kamal, Mohamed Ibrahim and Hala Zaid: Contributed analysis tools or data; Wrote the paper.

### Funding statement

This research did not receive any specific grant from funding agencies in the public, commercial, or not-for-profit sectors.

### Data availability statement

Data included in article/supplementary material/referenced in article.

### Declaration of interests statement

The authors declare no conflict of interest.

### Additional information

No additional information is available for this paper.
